# How to target membrane proteins for degradation: Bringing GPCRs into the TPD fold

**DOI:** 10.1016/j.jbc.2024.107926

**Published:** 2024-10-23

**Authors:** Boguslawa Korona, Laura S. Itzhaki

**Affiliations:** Department of Pharmacology, University of Cambridge, Cambridge, United Kingdom

**Keywords:** targeted protein degradation, TPD, membrane proteins, receptor, GPCR, lysosome, proteasome

## Abstract

We are now in the middle of a so-called “fourth wave” of drug innovation: multispecific medicines aimed at diseases and targets previously thought to be “undruggable”; by inducing proximity between two or more proteins, for example, a target and an effector that do not naturally interact, such modalities have potential far beyond the scope of conventional drugs. In particular, targeted protein degradation (TPD) strategies to destroy disease-associated proteins have emerged as an exciting pipeline in drug discovery. Most efforts are focused on intracellular proteins, whereas membrane proteins have been less thoroughly explored despite the fact that they comprise roughly a quarter of the human proteome with G-protein coupled receptors (GPCRs) notably dysregulated in many diseases. Here, we discuss the opportunities and challenges of developing degraders for membrane proteins with a focus on GPCRs. We provide an overview of different TPD platforms in the context of membrane-tethered targets, and we present recent degradation technologies highlighting their potential application to GPCRs.

Traditional drugs modulate protein activity, usually by inhibition such as blocking of an active site or binding site. However, we are now in the middle of a so-called “fourth wave” of drug innovation (1), characterized by the development of multi-specific medicines aimed at disease targets previously thought to be “undruggable.” By inducing proximity between two or more proteins, for example a target and an effector that do not naturally interact, these multi-functional molecules can have activities beyond those of conventional drugs. In particular, in recent years targeted protein degradation (TPD) strategies to destroy disease-associated proteins have emerged as an exciting pipeline in drug discovery (2) in both academia and industry (3, 4). A diverse range of strategies are used to harness the cell’s natural degradation machinery, including, but not limited to, PROteolysis TArgeting Chimeras (PROTACs) that recruit E3 ubiquitin ligases for proteasome degradation of targets, and LYsosome-TArgeting Chimeras (LYTACs) and Antibody-based PROTACs (AbTACs) that recruit the lysosomal machinery. Target degradation offers several advantages over target inhibition. First, as the ligand does not need to block a functional site, target degradation has the potential to tackle proteins that lack suitable pockets for small molecule inhibitors. Second, all functions of a multi-functional protein are inhibited by degradation. Third, sub-stoichiometric target engagement should ensure persistent event-driven effects that results in better pharmacological responses compared with the occupancy-based mechanism of conventional inhibitors. Consequently, TPD has been championed as a strategy that could expand the druggable proteome. Nevertheless, the requirement to modulate challenging proteins of interest (POIs) and to meet safety standards in clinical applications leaves room for further optimization and for new TPD concepts and platforms.

TPD of membrane and secreted proteins is still underrepresented in the literature, with efforts very much focused on intracellular targets. However, membrane proteins comprise roughly a quarter of the human proteome, and they are critical for cell-cell communication and signal transduction events that occur across the plasma membrane. G-protein coupled receptors (GPCRs) constitute a large majority of membrane proteins, and with 800 of them, this superfamily is the largest encoded in the human genome. GPCRs participate in the transmission of external signals to the inside of a cell and are implicated in over 80% of all transduction events that take place across the cell membrane (5). Hence, it is not surprising that dysregulation of membrane protein expression and function are associated with many diseases, as reflected in their domination of the drug market (6, 7, 8). Despite successes, there nevertheless remains the need to find new and improved drugs for membrane proteins (9), and TPD presents multiple opportunities and advantages compared with conventional inhibitors that may be able to address this need. Herein, we discuss the challenges and opportunities in developing degraders for membrane proteins with a focus on GPCRs. We provide an overview of different TPD platforms in the context of membrane targets, and we present recent cutting-edge degradation technologies highlighting their potential application to GPCRs. Finally, we highlight future perspectives and discuss new avenues that could be explored.

## Components for degrader design

Degradation technologies employ a variety of “warhead” modules—namely, a ligand that binds to the target POI—combined with a module that recruits the degradation machinery to bring the two into proximity and thereby drive target degradation. In the case of membrane-protein targets such as GPCRs, there is an additional consideration in the design of this bifunctional molecule, which is that many ligands bind to the extracellular domains of such targets but they need to bring the target to the degradation machinery located inside the cell. To this end, numerous innovative strategies are being developed using extracellular target-binding ligands combined with modules to drive internalization and subsequent degradation (mostly in lysosomes). Below we summarize recent developments in warhead and degradation modules and place them in the specific context of the intricate and complex biology and pharmacology of GPCRs.

## Target-binding ligands (warheads)

Currently, most TPD strategies use PROTACs composed of a small-molecule warhead that binds to the target linked to a small molecule that binds to an E3 ubiquitin ligase (referred to subsequently as E3). However, small molecule ligands present several challenges. First, a suitable pocket on the surface of the target is required, and hence many PROTACs to date are limited to targets that are already drugged. They are not well suited to targets such as scaffold proteins that function in mediating protein-protein interactions which typically have large and flat surfaces. Nevertheless, increasingly sophisticated computational approaches are enabling structural modeling and facilitated the screening and identification of new small molecule ligands (10). Second, small molecules can lack specificity leading to side effects that prevent them from progressing to the clinic. Third (discussed in more detail in the next section), E3s themselves are not readily amenable to liganding with small molecules, as they are mostly large, multi-domain, multi-subunit complexes. Thus, most PROTACs to date use just two E3s, cereblon (CRBN) and VHL (von Hippel–Lindau) despite there being hundreds of E3s in the human proteome and the need to harness more of them to broaden the range of targets and diseases accessible to TPD. Nevertheless, two orally bioavailable PROTACs - ARV-110 (NCT03888612) which targets the androgen receptor in prostate cancer, and ARV-471 (NCT04072952) which targets the estrogen receptor in breast cancer—have reached phase II clinical trials and many others are in progress through earlier phases (11).

Although GPCRs are the largest class of membrane protein drug targets (5), even the most advanced small molecules that modulate GPCR activity tend to suffer from a lack of specificity (7). Furthermore, over half of non-sensory GPCRs remain undrugged, of which orphan receptors without known endogenous ligands account for 27% (7). Targeting orthosteric sites, which tend to be conserved between GPCRs, can lead to undesired side effects, and, consequently, drugs that bind to allosteric sites are increasingly favored (5). The binding sites of allosteric GPCR ligands are generally in the extracellular and membrane domains, whereas the cell’s quality control machinery is located inside a cell; hence, as explained above, TPD strategies focus on internalization mechanisms to traffic the target to the lysosome for degradation. Agonist ligands could potentially be used as warheads for TPD, with the caveat that they might trigger downstream signaling rather than degradation and might need modification to avoid this issue.

Antibodies and antibody-like ligands are routinely used as binding moieties for membrane-embedded POIs and represent a major class of drugs. They are now being intensively explored as warheads for TPD. AbTACs and PROTABs (proteolysis-targeting antibodies) share a similar design, comprising a bi-specific antibody with one arm acting as the warhead to bind the POI and the other arm binding to the extracellular domain of a plasma membrane-localized E3. AbTACs were shown to drive the degradation of the programmed death-ligand 1 (PD-L1) and the epidermal growth factor receptor (EGFR) by harnessing the E3s RNF43 (RING-finger protein 43) and ZNRF3 (zinc- and RING-finger 3) (12, 13). PROTABs likewise harness transmembrane E3s and have been used to drive the degradation of insulin-like growth factor 1 receptor (IGF1R), HER2 (human epidermal growth factor receptor), and PD-L1 (14). Single-domain antibodies, such as nanobodies, have similar key attributes to conventional antibodies such as high affinity and specificity, but their smaller size and simpler composition provide additional benefits in some applications. Moreover, nanobodies can bind to small interfaces that are not suited to antibodies, and some are able to discriminate between different functional states of target proteins or closely related family members, and they are being used very effectively for TPD. An example is the RHOB-GTPase, for which a cell-based screen led to the identification of an “intrabody” (intracellular single-domain antibody) that has high specificity for the GTP-bound state. This nanobody was used to construct a “bioPROTAC”—a chimeric E3 in which the natural substrate-binding domain is replaced by a binder to the POI—which was shown to promote RHOB-GTPase degradation (15). Another example is a highly selective “monobody” against the active state of cancer-associated KRAS mutants that, when converted to a VHL-based bioPROTAC, selectively degraded the KRAS mutants, providing a more extended suppression of mutant KRAS activity than that achieved by the monobody alone (16). Efforts to reduce the size of antibodies and engineer them for cell permeability led to the creation of cell-penetrating nanobody-based degraders of BCL11A composed of an engineered BCL11A-specific nanobody fused to one of a number of different E3 adaptors (17). This study outlines the power of protein-based ligands like nanobodies—the nanobody was highly specific for BCL11A and did not bind the closely related paralog BCL11B, a feat that would be hard to achieve with a small molecule. It should be noted that not only the affinity of the antibody but also the presentation of its epitope to the POI will affect its potency (12), and modifications of antibody scaffolds to alter their flexibility and valency can enhance the efficiency of degradation (12, 14).

Peptide ligands, typically 10 to 20 amino acids in length, have also been used as warheads for TPD. Rapid advances in *in silico* modelling and library screening technologies have made it easier to develop binding peptides (16, 18, 19, 20, 21, 22), and peptide-based PROTACs should enable access to targets that are hard to drug with small molecules (21). However, a major drawback of peptide drugs is their low membrane permeability, which limits oral bioavailability and tissue distribution. To overcome this challenge, researchers have used cell-penetrating peptides (CPPs) and nanoparticles as delivery systems (23). Some commonly used CPPs originate from viral proteomes, for example Tat from human immunodeficiency virus-1 (HIV-1) and VP22 from herpes simplex virus-1 (HSV-1), as well as several novel sequences recently identified from SARS-CoV-2 (24). Interestingly, some of these CPPs feature tumor-specific motifs, which can be potentially utilized in cancer therapies (24).

The majority of existing peptide therapeutics against GPCRs are modifications of naturally occurring peptide ligands (25), including the groundbreaking work on insulin from the 1920s. These ligands often have exceptional pharmacodynamic properties including high affinity, selectivity, and potency, and they are well tolerated and have minimal side effects, although strategies are needed to extend their otherwise short half-lives (25). It is likely that the challenges associated with the transient responses and rapid turnover of peptides composed of natural amino acids may potentially be mitigated by using event-driven TPD instead of conventional occupancy-driven approaches. Full-length (or close to full-length) natural signaling peptides of up to 40 amino acids are also experiencing a revival in GPCR drug discovery, and many natural and modified peptides targeting GPCRs have been approved for clinical use and more are in clinical trials (25). Another class of GPCR ligands is biased agonists that direct the receptor to either G-proteins or β-arrestins to modulate specific downstream signaling events or promote receptor desensitization, respectively. For example, β-arrestin-biased melanocortin four receptor (MC_4_) agonists are promising drugs for the treatment of obesity (26). Both peptides and small-molecule biased agonists have been identified to date.

GPCRs should not be viewed as monomeric entities. There is overwhelming evidence that *in vivo* some G-protein receptors form homo- or heteromeric complexes (27). Co-existence and cooperation of these GPCRs is a prerequisite to modulate one another’s activity and to ensure their proper functions. The GPCRs oligomerization issue is still therapeutically unexplored. Dual agonist peptides that activate two different GPCRs, and multivalent ligands (small molecule and peptide-based) for specific heteromers are also under development (28, 29).

Given the potential of peptides to expand the druggable proteome, there have been intensive efforts to improve their properties such as stability *in vivo*. Strategies include chemical modifications and crosslinking, unnatural amino acids, and D-amino acids, all of which increase their resistance to proteolysis (30, 31). In some cases, these modifications are introduced into the crosslinker rather than the peptide (32). Cross-linking of peptides (also referred to as ‘stapling’) induces a binding-competent conformation such as an alpha-helix and thereby increases the binding affinity for the target as well as enhancing the stability (33, 34, 35). Cross-linking can also increase the cell permeability, although there is still no common set of rules governing the observed effects. A stapled peptide used to target the programmed cell death-1/programmed cell death-ligand 1 (PD-1/PD-L1) exhibited enhanced affinity and selectivity and more pronounced degradation of PD-L1 compared with the unstapled version (36). Stapled peptides that bind to GPCRs, such as the modified orexin ligand for the orexin receptor and stapled peptide oxyntomodulin (OXM) analogues that display dual agonist activity against GLP-1R and GCGR, have also been explored (37, 38). One interesting area is lipidated “pepducins” derived from the sequences of intracellular loops of GPCRs. Pepducins are able to penetrate the cell membrane and, thereby, reach allosteric sites with the aim of stabilizing GPCRs (25). Another route to extending the half-lives of short-lived peptides is to conjugate them to single-domain antibodies such as the single-domain albumin antibody (AlbudAb). Lastly, inducible and reversible self-assembly peptides, such as OXM that forms nanofibrils that dissociate *in vivo* to release active peptide extending its half-life and simultaneously activity, also have tremendous potential (39). In this way so-called ‘aptamers’ can be developed to specifically and tightly bind to a wide range of target proteins including membrane-embedded entities. In summary, peptides offer low-cost warheads for TPD that exhibit high biological activity and specificity (21, 40). Other biomolecules, such as single-stranded DNA or RNA, are also being explored for use as warheads in TPD. In this regard, affinity screening of random oligonucleotide sequences by exponential enrichment (SELEX) technology is a promising tool for the identification of so-called “aptamer” ligands for TPD of membrane proteins (41).

## Degradation-recruiting ligands

### Ubiquitination-based strategies

Harnessing E3 ubiquitin ligases is the paradigm of PROTACs, the best validated TPD approach to date. As discussed above, one significant limitation is that most E3s are complex, multi-domain, and (often) multi-subunit entities that lack natural small molecule ligands and have been difficult to ligand to date. Consequently, many of the PROTACs in the public domain harness just two E3s—VHL and CRBN—even though it is likely that other E3s may be needed to broaden access to targets, enhance cell/tissue specificity, and avoid complications such as drug resistance. Furthermore, the majority of E3s reside in the cytosolic compartment, making them less intuitively well-suited for targeting membrane POIs for degradation when combined with extracellular warheads. Transmembrane E3s with extracellular domains are attractive candidates to be exploited in combination with antibody or peptide-based warheads. AbTACs and PROTABs are bi-specific antibodies in which one arm acts as the warhead to bind the POI and the other arm binds to the extracellular domain of a transmembrane E3. In this way ZNRF3, RNF43, RNF130, RNF133, RNF149, and RNF150 were co-opted for TPD of PD-L1, EGFR, and IGF1R (12, 13, 14), and three transmembrane E3s (RNF128, RNF130, and RNF167) have recently been co-opted using a nanobody-based platform named REULR (Receptor Elimination by E3 Ubiquitin Ligase Recruitment), thereby further expanding the membrane TPD toolkit (42).

Cytosolic E3 ubiquitin ligases, VHL and CRBN, which have been extensively used for PROTACs against cytosolic POIs, have also been harnessed for cell-surface POIs, including EGFR and androgen receptor, “from the inside out” (*i.e.*, using warheads that bind to the POI’s intracellular domains) (43). And, although it seems counterintuitive to make PROTACs combining ligands for cytosolic VHL and CRBN with warheads for the extracellular domains of cell-surface POIs, given that the two moieties, such PROTACs have been shown to be capable of degrading of two membrane receptors. The first comprised the α_1_-adrenoceptor (α_1_-AR) subfamily antagonist prazosin linked to a ligand for the CRBN (44). The second comprised an orthosteric ligand of the G protein-coupled estrogen receptor (GPER) linked to a ligand for the VHL (45). It is hard to explain the activity of hetero-bifunctional molecules that have an extracellular target-recognition moiety and an E3 recruiter that needs to face the cytosolic space. It may be that they act inside the cell during POI biogenesis or trafficking.

E3s implicated in the regulation of GPCR signaling, such as CHIP, Nedd4, Mdm2, and c-Cbl, merit attention due to their proven ability to eliminate this class of membrane proteins (46). Of note, membrane protein substrates of CHIP are usually conjugated with K63-linked polyubiquitin chains (47, 48, 49), and cytosolic CHIP substrates are usually conjugated with K48-linked ubiquitin chains (50), whereby the former direct the target to the MVB (multivesicular bodies) pathway for lysosomal degradation (51, 52) and the latter for proteasomal degradation.

**Figure 1:**
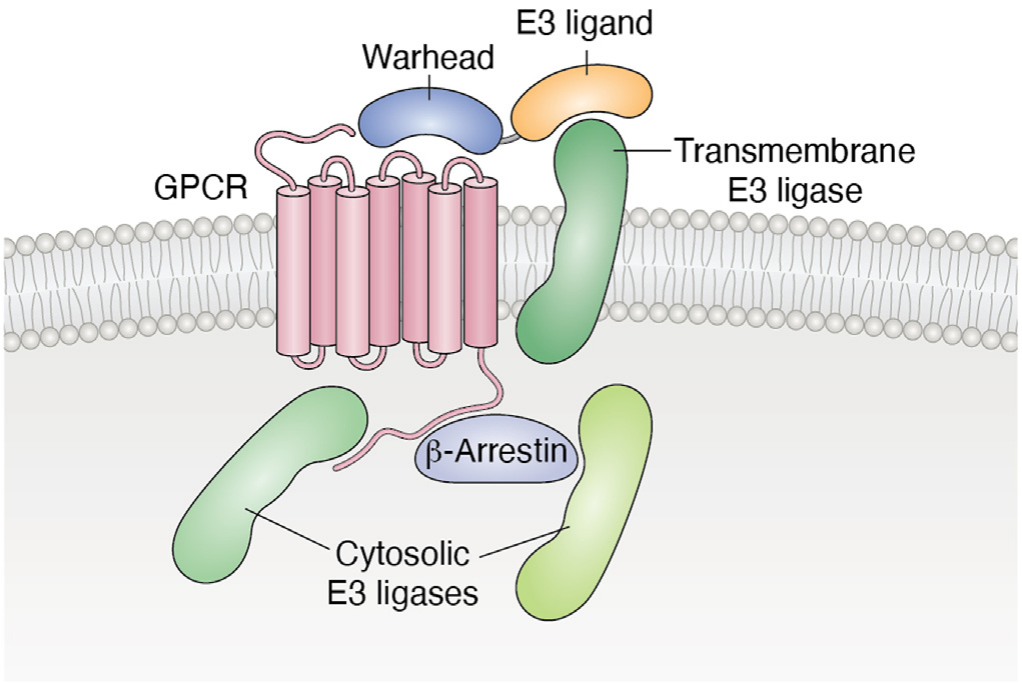
**Schematic showing how E3 ubiquitin ligases can be exploited for targeted degradation of transmembrane proteins such as GPCRs.** Targeted degradation can be achieved using hetero-bifunctional molecules combining a warhead (in *blue*) to bind to the extracellular face of the GPCR and a ligand (in *orange*) for a transmembrane E3 ubiquitin ligase. Cytosolic E3 ubiquitin ligases, implicated in the regulation of GPCR signaling either directly by binding to the intracellular face of the receptor or indirectly *via* β-arrestins, could also be co-opted for targeted protein degradation.

To improve the efficiency of PROTACs, both the expression profile of the E3 as well as its tissue-dependent distribution must be taken into consideration (20). Equally important are the subcellular localizations of the target and the E3 (53). In the case of GPCRs, an E3 may interact directly with and ubiquitinate residues located in an intracellular loop or tail of a GPCR or indirectly *via* β-arrestins as adaptors for the E3s (54) (Fig. 1). Ubiquitination of a GPCR drives its recruitment into the ESCRT (endosomal-sorting complex required for transport) pathway (46). The balance of accessible GPCRs is regulated by the interplay between activities of E3s that promote degradation and deubiquitinating enzymes (DUBs) that remove the ubiquitin moieties leading to receptor recycling and insertion in the plasma membrane. Interestingly, arrestin-domain containing proteins (ARRDCs) proteins appear to direct their substrates for degradation. Arrestin-related Domain-containing Protein-3 (ARRDC3) has been implicated in reducing 2-adrenergic receptor levels in mice and in human cells (55, 56, 57, 58). Arrestin-related ARRDC3 recruits the E3 Nedd4 (neural precursor cell expressed, developmentally down-regulated-4) to reduce receptor levels (59), and two C-terminal PPXY motifs of ARRDC3 have been shown to be important for the interaction. Thus, a bifunctional degrader with one arm composed of a PPXY motif to recruit Nedd4 and another to bind a GPCR could be an effective TPD approach.

As mentioned above, ubiquitin conjugation to ligand-stimulated GPCRs targets these receptors to lysosomes *via* the ESCRT pathway (60). GPCRs can be ubiquitinated *in trans* following activation of another receptor, or conversely, a GPCR in a dimeric complex is capable of undergoing co-ubiquitination and co-degradation of the non-activated binding partner (61). Such a phenomenon was revealed to alter the cell surface expression of the type 1 parathyroid hormone (PTH) receptor upon biased agonist stimulation. The activating ligand results in PTH receptor ubiquitination prior to the deubiquitination process, and subsequently the receptor is recycled back to a plasma membrane, whereas the non-activating ligand undergoes sustained ubiquitination and subsequent degradation. Thus, ligand-stimulated PTH sorting can be attributed to the balance of ubiquitination and deubiquitination (62).

Although the lysosome is the key machinery for GPCR destruction, clearance *via* the proteasome and autophagy is also crucial in the regulation of these receptors (54). Additionally, GPCRs have been found in exosomes and microparticles indicating diversity of their trafficking and signaling patterns. This highlights the complexity of GPCR regulation in the cell and opens new perspectives for TPD design.

### “Escorted” degradation

**Figure 2:**
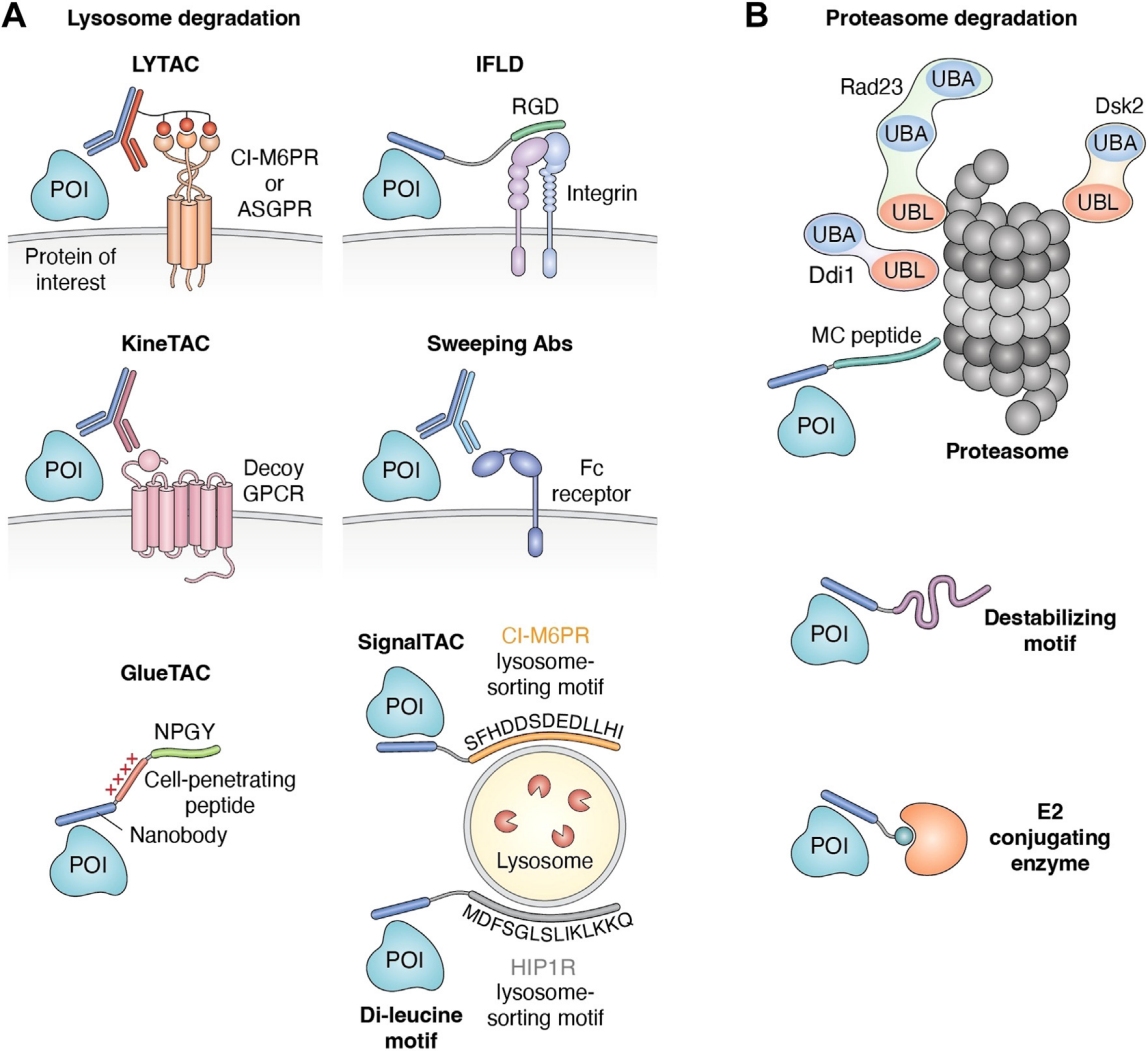
**Schematic showing other strategies for targeted degradation of transmembrane proteins.** Design of hetero-bifunctional molecules comprising a POI-binding motif (*blue line*) combined with different degradation-recruiting motifs. *A*, lysosome-mediated degradation. Lysosome-targeting chimeras (LYTACs) utilize cell surface receptors CI-M6PR (cation-independent mannose-6- phosphate receptor) or ASGPR (asialoglycoprotein receptor) to target a protein of interest (POI) to lysosomes for degradation. Integrin-facilitated lysosomal degraders (IFLDs) exploit the integrin ligand RGD to internalise a POI for lysosome-mediated destruction. Cytokine receptor-targeting chimeras (KineTACs) are bispecific antibodies comprising a POI-binding arm (in *blue*) and a cytokine arm as a ligand for its cognate cytokine receptor (a decoy GPCR). Sweeping antibodies (Abs) use a variety of Fc receptors to degrade a POI in a pH-dependent manner. Covalent nanobody-based degrading chimeras (GlueTACs) are composed of a nanobody that binds covalently to a POI linked to a positively charged cell-penetrating peptide and the lysosome-sorting motif NPGY from LDLR. Signal-mediated lysosome-targeting chimeras (SignalTACs) target a POI for lysosomal degradation through the lysosome-sorting motif SFHDDSDEDLLHI from CI-M6PR. Di-leucine lysosome-sorting motifs (*e.g.*, MDFSGLSLIKLKKQ from HIP1R) can be used to target a POI to the lysosome for degradation. *B*, proteasome-mediated degradation. *Top*: recruitment to the 26S proteasome *via* shuttle proteins Rad23, Dsk2, and Ddi1, or *via* a short proteasome-binding peptide: Shuttle proteins contain ubiquitin-like domains (UBL) that are recognised the proteasome ubiquitin-associated domains (UBA) to bind to ubiquitinated substrates; MC peptides were identified with high affinity for proteasomal subunit PMSD2. *Middle*: destabilizing motifs, such as PEST sequences (rich in proline (P), glutamic acid (E), serine (S), and threonine (T)) can be used as ligands for proteasomal degradation. *Bottom*: recruitment of an E2 ubiquitin-conjugating enzyme to direct a POI for degradation.

Matrix-residing recycling receptors with readily triggered internalization mechanisms are being exploited to drive the ‘escorted’ degradation of membrane and secreted proteins. Lysosome-targeting chimeras (LYTACs), developed by the Bertozzi group, are designed to harness glycan-based recycling receptors such as the cation-independent mannose-6-phosphate receptor (CI-M6PR) or the liver-specific asialoglycoprotein receptor (ASGPR) (Fig. 2*A*). Internalization *via* CI-M6PR and ASGPR has been exploited therapeutically elsewhere, for example for the delivery of exogenous lysosomal enzymes (for lysosomal storage disorders) (63) and the delivery of siRNA to the liver (64). LYTACs comprise a module (usually an antibody) that binds to a POI conjugated with the glycan ligands poly(M6Pn) or tri-GalNAc that bind to the abovementioned receptors to transport the POI to lysosomes for degradation (65). It is likely that LYTACs work stoichiometrically rather than catalytically, and another limitation is the need to identify and synthesize the requisite antibody-glycan modification for efficient degradation. ASPGR-mediated internalization is also exploited in bifunctional small molecule degraders named MODE-A developed by the Spiegel lab (66).

The integrin αvβ3 has been widely used for the targeted delivery of anti-cancer drugs, due to its overexpression in various tumor cells, and it is now being exploited for TPD of membrane proteins (67). It is particularly attractive because of the simple three-residue peptide ligand (sequence RGD). Integrin-facilitated lysosomal degraders (IFLDs) are bifunctional molecules comprising a cyclic integrin recognition motif connected *via* a linker to a POI binder (Fig. 2*A*). Formation of a ternary complex between POI and integrin on the cell surface leads to endocytosis followed by lysosomal degradation of the POI. For example, IFLDs were shown to efficiently mediate the lysosomal clearance of PD-L1 (67).

Natural recycling receptors for cytokines and growth factors are also being exploited for TPD. For example, KineTACs (cytokine receptor-targeting chimeras) developed by Well’s team, are bispecific antibodies composed of a cytokine arm as a ligand for the cognate cytokine receptor and a POI-binding arm (Fig. 2*A*) (22). The cytokine receptor is referred to as a “decoy” recycling receptor. Decoy receptors recognize certain inflammatory cytokines with high affinity and specificity but are structurally incapable of signaling or presenting the agonist to signaling receptor complexes. They thereby act as a molecular trap for the agonist and for signaling receptor components. KineTACs have been developed exploiting various different cytokines and their receptors, specifically CXCL12 -CXCR7, CXCL11-vMIPII, and the interleukin2-IL2 receptor. The versatility of receptors provides the opportunity to tailor KineTACs to tissue-specific contexts that may broaden their therapeutic applications and impact on treatment outcome.

So-called “sweeping antibodies” co-opt various Fc (fragment crystallizable) receptors to internalize POIs and provide an alternative degradation modality (19, 68, 69). Sweeping antibodies are engineered to have improved binding to Fc recycling receptors FcRn (neonatal Fc receptor) or FcγRIIb and pH-sensitive CDRs (complementary-determining regions) that bind to the POI at neutral pH but release it in the acidic endosomes after internalization of the complex. This results in delivery of the POI to the lysosome and subsequent degradation, and the antibody bound to the Fc receptor is recycled to the cell surface where it can target further POI molecules. Sweeping antibodies have been used for targeting degradation of proteins such as the IL-6 receptor (19) and multiple different soluble antigens. There are, however, some challenges with this technology. First, histidine residues are engineered into the CDRs to make them pH sensitive, but finding site suitable for mutation that do not disrupt POI binding at neutral pH is not straightforward. Second, Fc receptors are not uniformly expressed, limiting degradation in all tissues. Third, sweeping antibodies compete with natural immunoglobulins, which are present in large excess and so limit their access to Fc receptors.

### Degradation *via* lysosomal adaptors

Alternatively, lysosomal degradation can be executed by using short peptides that act as lysosome-sorting motifs. For example, covalent nanobody-based degrading chimeras (GlueTACs) comprise a single polypeptide chain composed of a nanobody conjugated to a reactive unnatural amino acid to bind covalently to the POI (termed ‘GlueBody’) linked to a cell-penetrating peptide sequence (a poly-D-arginine sequence) and the lysosome-sorting motif NPGY from the low-density lipoprotein receptor (LDLR) (Fig. 2*A*). The bound POI is then transferred to the lysosome for degradation (70). The SignalTAC (signal-mediated lysosome-targeting chimera) platform was likewise capable of targeting membrane proteins for degradation using a fusion of a lysosome-sorting motif from CI-M6PR and peptide- or nanobody-based POI-binding modules (71). Another study dissected the function of the protein HIP1R, which binds to PD-L1 and transfers PD-L1 to the lysosome for degradation. A di-leucine lysosome-sorting motif (MDFSGLSLIKLKKQ) and a PD-L1-binding motif were identified in HIP1R, and a chimeric peptide comprising a fusion of these two sequences was then shown to be capable of driving the degradation of PD-L1 in tumor cells (72). The use of short lysosome-sorting motifs needs to be validated for GPCRs, but the potential of such ubiquitin-independent TPD platforms is tremendous. They eliminate the need for lysine residues on the POI and may be easier to design (given that, compared with a PROTAC, there could be less stringent requirements for a specific orientation induced between the POI and the degradation machinery).

Finally, the P2Y_1_ purinergic receptor internalizes to endocytic vesicles upon ADP stimulation. A conserved YPX3L motif (where X is any amino acid) within the second intracellular loop of P2Y_1_ interacts with ALG-interacting protein X (ALIX), a multivalent adaptor protein. This interaction mediates sorting at late endosomes into intraluminal vesicles (ILV) of the multivesicular bodies (MVB) to facilitate degradation, bypassing the requirement for ubiquitination for ESCRT engagement (73). This work demonstrates that some actively stimulated receptors can undergo lysosomal degradation *via* ubiquitin-independent pathways. ALIX and other adaptor proteins such as the GPCR associated sorting protein-1 GASP-1 can promote lysosomal sorting, expanding the pool of the ubiquitin-independent ESCRT recruiters (74), and it would be interesting to explore these types of degraders for other GPCRs that have the YPX3L sequence.

## Future perspectives

Most substrates require ubiquitination by E3s to be targeted to the proteasome. However, direct targeting of the proteasome is also possible and would have the advantage of not being subject to the counteracting effects of deubiquitinating enzymes. Proteasome “shuttle” proteins, such as Rad23, Dsk2, and Ddi1, comprise a ubiquitin-associated domain (UBA) and a ubiquitin-like domain (UBL), where the former binds to a ubiquitinated substrate and the latter binds to proteasome receptors Rpn1/S2/PSMD2, Rpn10/S5a, and Rpn13/Adrm1 (75, 76, 77) (Fig. 2*B*). The UBL domains of these shuttles has been exploited to transfer POIs directly to the proteasome (78). The recently discovered MC peptide ligands for the proteasome subunit PSMD2 were also shown to enable target degradation (79) (Fig. 2*B*). The C-terminus of ornithine decarboxylase (cODC) mimics the polyubiquitin chain for recognition by the proteasome (80, 81), and degraders derived from known destabilizing motifs - sequences rich in proline (P), glutamic acid (E), serine (S), and threonine (T) (PEST) - such as cODC could potentially be used for TPD of membrane proteins (Fig. 2*B*). Most PROTACs to date have co-opted E3 ubiquitin ligases, but E2 ubiquitin-conjugating enzymes could potentially also be co-opted for TPD (82, 83) (Fig. 2*B*).

**Figure 3:**
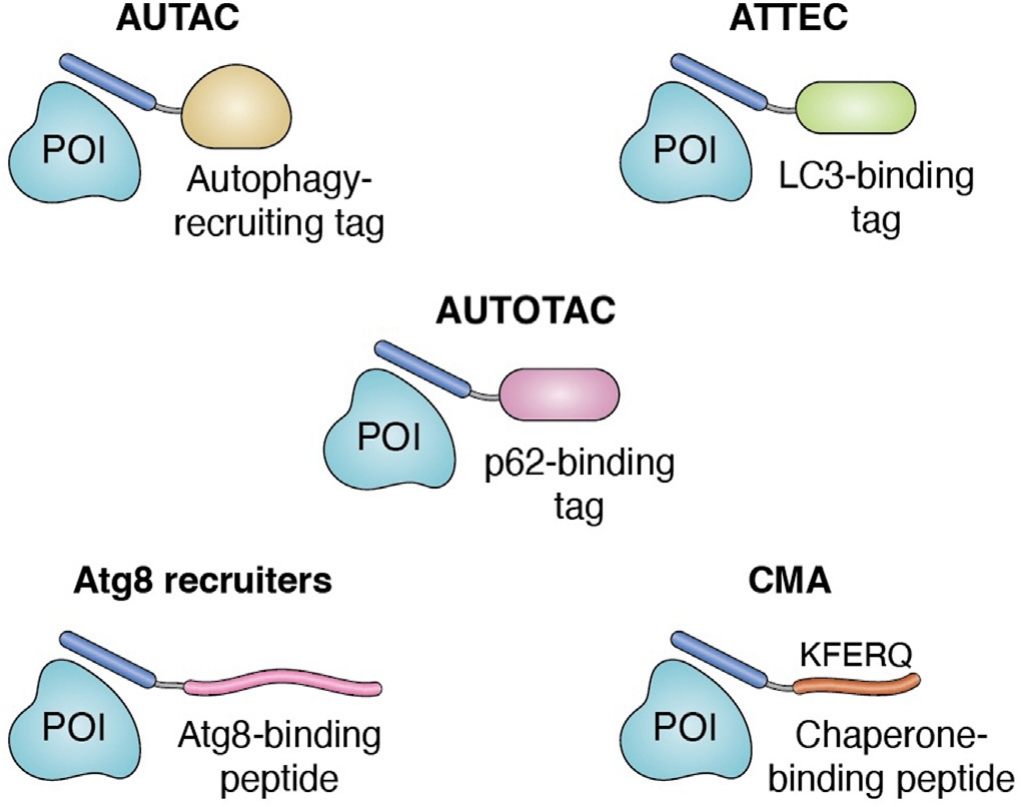
**Schematic of different designs of autophagy-harnessing degraders.** Design of hetero-bifunctional molecules comprising a POI-binding motif (*blue oval*) linked with one of the following autophagy-recruiting motifs: Autophagy-targeting chimeras (AUTACs) comprise a small molecule ligand for a POI linked to a small molecule autophagy-recruiting tag. Autophagosome tethering compounds (ATTECs) comprise a small molecule ligand for a POI linked to a small molecule ligand for the phagophore (autophagosome precursor) protein LC3. AUTOTACs comprise a small molecule ligand for a POI linked to a small molecule ligand for the autophagy cargo receptor p62/SQSTM1. Short peptide sequences can be used to recruit members of the Atg8 family of autophagy proteins. Chaperone-mediated autophagy (CMA): KFERQ-like sequences exposed on the surface of a damaged or misfolded protein are recognised by the cytosolic molecular chaperone Hsc70 and targeted to autophagy-mediated degradation.

Alternatively, autophagy pathway components could be co-opted for GPCR degradation (11, 84). Many hetero-bifunctional molecules have been developed to promote the formation of POI-specific autophagosomes and subsequently drive the degradation of the POI *via* lysosomes (Fig. 3). Inspired by the role of *S*-guanylation in targeting biomolecules for autophagy, autophagy-targeting chimeras (AUTACs) were created consisting of a guanine tag linked to a POI-binding ligand. In autophagosome-tethering compounds (ATTECs), a small molecule ligand for the phagophore (autophagosome precursor) protein LC3 is linked with a POI-binding ligand (85, 86). And in AUTOTACs, a ligand that binds the autophagy cargo receptor p62/SQSTM1 is linked with a POI-binding ligand (87). Short peptides that bind specific members of the Atg8 family of autophagy proteins have been optimized and engineered with enhanced potency by fusing then to oligomerization domains (88), and most recently, an artificial intelligence–based structure prediction tool was used to identify Atg8-binding motifs, which could enable the expansion of the autophagy degrader toolbox (89). These autophagy-promoting peptides could be used to create peptide-based autophagy-targeting chimeras for degradation of a POI. Chaperone-mediated autophagy (CMA) leads to selective lysosomal degradation of damaged or partially misfolded proteins, whereby the cytosolic molecular chaperone heat shock cognate 71 kDa (Hsc70) recognizes a characteristic KFERQ-like motif that is present in ∼40% of human proteins (90). The resulting substrate-chaperone complex binds to the lysosome-associated membrane protein type 2A (L2A) that initiates multimerization of L2A into a translocation complex for lysosomal degradation (91, 92). Thus, a POI could potentially be targeted for lysosomal degradation by bridging with a KFERQ motif.

Proteins can be regulated at many stages of their biogenesis, starting from their encoding of nucleic acids. RiboTACs (ribonuclease targeting chimeras) provide a route to target RNA for destruction (93). Nature-inspired compounds were shown to bind RNA structures and in combination with the RNA recycling enzyme prevent target proteins from being synthesized. Moreover, RiboTACs can eliminate target RNA regardless of the cellular localization of the biosynthesis product, and thus RNA-degrading modalities could be an attractive approach for GPCRs and extracellular proteins. Notably, RNA-binding proteins modulate GPCR expression at the posttranscriptional level (94). With in-depth knowledge of a specific interaction between cytosolic RNAs and the mRNA of a given GPCR, the RiboTACs appear to have great potential.

**Figure 4:**
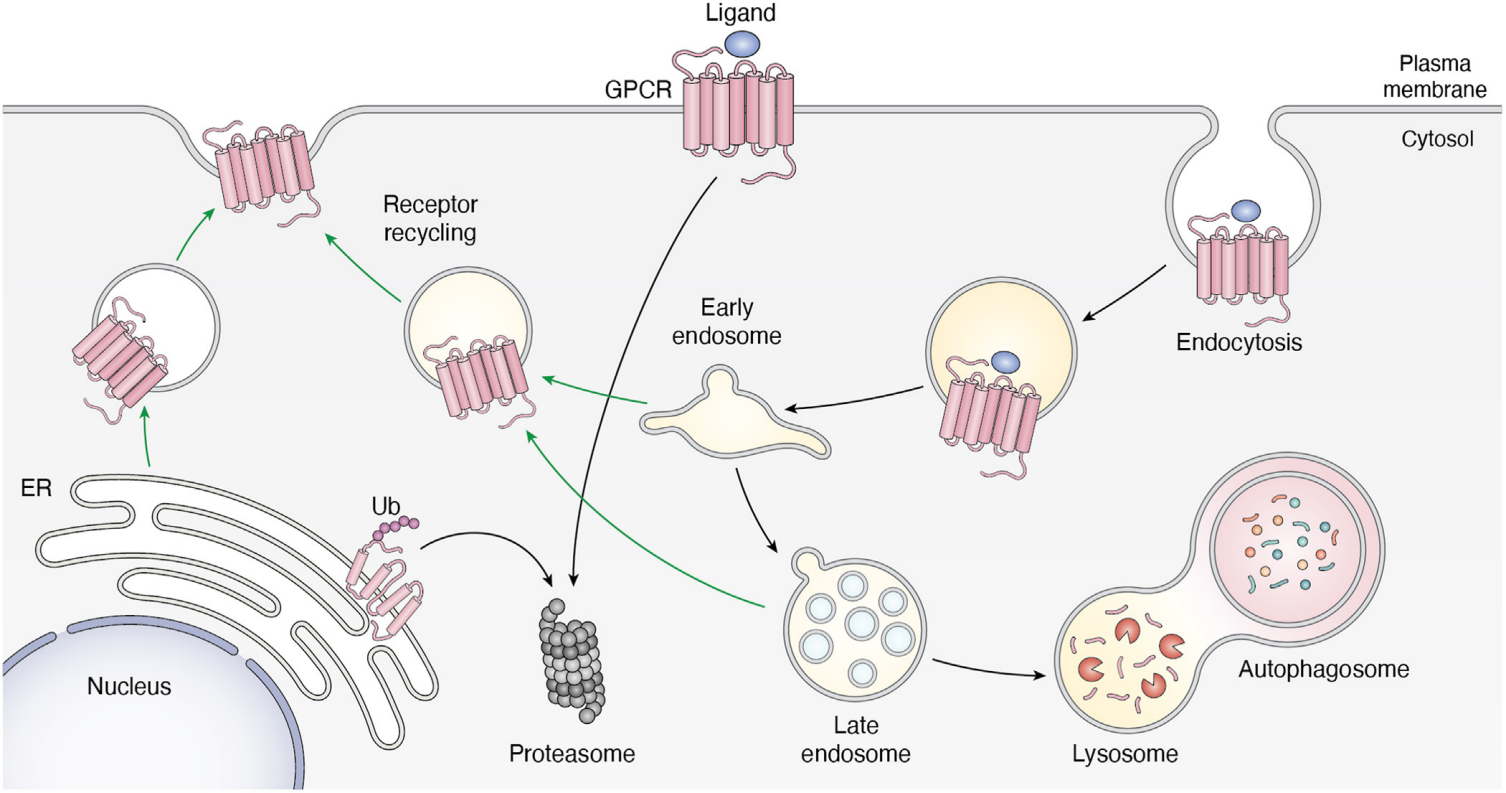
**Endogenous pathways of receptor degradation.** Ligand-stimulated GPCRs upon endocytosis are internalized and then are subjected to endocytic sorting to either recycle them back to a cell membrane or to direct them for lysosomal degradation. Some GPCRs are directed to the proteasome for degradation. ER-associated degradation pathway (ERAD) directs newly synthesised receptors to the proteasome for degradation, whereas correctly folded receptors are trafficked to the plasma membrane.

To most effectively target GPCRs it is imperative to explore all aspects including biogenesis, activation, internalization, recycling, and destruction (Fig. 4). The endoplasmic reticulum (ER) protein quality control mechanism tightly regulates the trafficking of newly synthesized GPCRs from intracellular compartments to the cell surface (95). Receptors that fail to fold or misfold, including disease-associated mutant variants, are subjected to ER-associated degradation (ERAD) (96, 97, 98, 99) (Fig. 4). This raises the question of whether ERAD, with at least 10 ER membrane-bound E3s could be explored for GPCR elimination (100). Multiple quality checkpoints exist to ensure proper protein function that is especially challenging for recycling GPCRs. ER-phagy (reticulophagy), the degradation of portions of the endoplasmic reticulum within lysosomes or vacuoles, prevents the failure of protein quality control caused by perturbations of the ER homeostasis and its dysfunction. Coordinated crosstalk between the transmembrane E3 TRIM13 and the autophagy cargo receptor p62 initiates ER turnover (101). It is also important to note the lipid microenvironment as a key effector of GPCRs stability and activity. GPCRs localize and cluster in lipid rafts that contain a unique lipid composition (102), and perturbations can destabilize GPCRs and cause their rapid degradation.

In summary, there have been exciting efforts over the last 5 years in both academia and industry to shift the focus of TPD to membrane proteins, in particular harnessing nature’s way of internalizing and destroying these proteins. We expect in the next 5 years that we can exploit the extensive knowledge of GPCR biology and pharmacology to direct TPD strategies at this important membrane protein class.

## Conflict of interest

The authors declare that they have no conflicts of interest with the contents of this article.
